# Characterizing the Tensile Strength of the Fabrics Used in Firefighters’ Bunker Gear under Radiant Heat Exposure

**DOI:** 10.3390/polym14020296

**Published:** 2022-01-12

**Authors:** Nur-Us-Shafa Mazumder, Sumit Mandal, Robert J. Agnew, Adriana Petrova, Lynn M. Boorady, Guowen Song

**Affiliations:** 1 Department of Design, Housing and Merchandising, Oklahoma State University, Stillwater, OK 74078-5061, USA; shafa.mazumder@okstate.edu (N.-U.-S.M.); adriana.petrova@okstate.edu (A.P.); lynn.m.boorady@okstate.edu (L.M.B.); 2 Fire Protection and Safety Engineering Technology Program, Oklahoma State University, Stillwater, OK 74078-5061, USA; rob.agnew@okstate.edu; 3 Department of Apparel, Events & Hospitality Management, Iowa State University, Ames, IA 50011-2100, USA; gwsong@iastate.edu

**Keywords:** fire protective textiles, sweat moisture, fabric strength, radiant heat, textile properties

## Abstract

More than 60,000 firefighters’ injuries were reported by the National Fire Protection Association in the U.S. in 2019. Inadequate protection by bunker gear could be a reason for most of the injuries. Firefighters repeatedly encounter thermal hazards due to their job responsibilities. Degradation could occur on bunker gear fabric during thermal exposure. It has been found that the presence of moisture affects performance as well, which may come from wearers’ sweat. Proper evaluation of the tensile strength of the fabrics used in bunker gear could provide information essential for maintenance the overall integrity of the gear. An evaluation of the tensile strength of fabrics when exposed to 10, 15, and 20 kW/m^2^ radiant heat flux in the presence of moisture is reported. In each fabric system, a total of sixty-four different samples were prepared for four different types of fabric and four levels of moisture which were exposed to three different radiant heat flux for five minutes. Heat flux and moisture levels have significant impact on tensile strength. The effect of moisture on tensile strength in a three-layered fabric system is higher than that for a single layer fabric. An understanding of the impact of heat and moisture on fabric strength has been achieved.

## 1. Introduction

Firefighting is considered a hazardous occupation because of the environment in which firefighters work. Fire departments in the U.S. responded to 1.3 million fires in 2019, and there is always the risk of death and injury for on-duty firefighters [1,2]. Based on the survey “United States Firefighter Injuries in 2019”, the National Fire Protection Association (NFPA) estimated that in 2019 over 60,000 firefighters’ injuries occurred in the line of duty. Around 40% of these injuries happened at the fire-ground, the area where firefighting operations are carried out [1,3]. In total, 48 firefighters died while on duty in the U.S. in 2019 [4]. Improved protective clothing can minimize the risk of injuries to firefighters [5,6].

Thermal protective fabrics are exposed to various thermal hazards (i.e., radiant heat, steam, hot fluids, etc.) during their lifetime. The outer layer of the fabric comes into direct exposure with these hazards every time bunker gear is worn to a fireground. These hazards might cause polymer chain scission of the fiber which may lead to a change in tensile strength. This may change the integrity of the clothing, a degradation which is rarely considered. However, the loss of protective performance due to the loss of tensile strength, which is caused by polymer degradation of the fiber of the outer layer, might not be detected visually until the damage is extreme. The reduction in thermal protective performance due to the potential strength loss of the outer layer fabric not only has an economic cost, but also it is related to the safety of the firefighters. Therefore, it is very important to know how the outer layer is affected as it is exposed to various thermal hazards over time [7,8].

It has been found that performance of the fabric is affected by moisture accumulated inside the fabric [9,10,11,12,13,14,15,16,17,18]. Moisture negatively affects the performance of the fabric most severely when lower amounts of moisture are added. When exposed to radiant heat, wetted samples provided more thermal protection than dry samples [15]. Depending on the heat intensity, heat transfer may be increased or decreased by the moisture in the clothing system. Under high heat flux flame exposure, internal moisture increases the heat transfer. This scenario is reversed for the low heat flux flame exposure. Under low heat flux exposure, internal moisture has a tendency to decrease the heat transfer for a two-layered wildland firefighters’ clothing system when the inner layer is completely soaked [10].

The effects of repeated exposure to heat of personal protective gear have often been studied [7,8,19,20,21,22]. Variables included intensity of heat flux, time, and frequency of exposure, among others. High heat exposure causes more loss of tensile strength compared to that caused by low heat exposure. Duration of exposure effects tensile strength loss [7]. Fire protective gear which when subjected to low radiant heat exposure has been evaluated [8]. Heat intensity has significant effects on the mechanical performance of the fabrics. However, the thermal protective performance did not change considerably as a consequence of changes in the mechanical properties [8]. The effects of flash fire on the mechanical properties of single layer thermal protective fabrics have been examined [19]. Similar kinds of results were found; the heat flux significantly affected the mechanical properties of the thermal protective fabrics [19].

The strength loss of the outer layer due to repeated thermal exposure could lead to the disintegration of the clothing system. This ultimately could contribute to the causes of injury to firefighters. The change of strength of the fabrics used in bunker gear due to radiant heat exposure has been extensively examined [7,8,19,20,21,23,24,25,26,27,28,29,30]. However, a determination of strength loss of the outer layer after being exposed to heat when the fabric is in moist condition has not been carried out. The strength loss of the outer layer fabrics used in bunker gear under different heat exposures is reported. Moisture exists in the inner layer due to fire fighter’s sweating, which also has an impact on the overall integrity of the apparel. Therefore, strength loss for fabric under different heat exposures in dry and wet conditions has been evaluated. A statistical modeling approach has been used to analyze various factors affecting the change of tensile strength when exposed to radiant heat in the presence of moisture.

## 2. Materials and Methods

In order to provide protection from the high heat encountered at a fireground firefighter bunker gear is constructed with single and/or multiple layers of high-performance fabrics. The bunker gear consists of single or multiple layer of high-performance fabrics. High-performance fabrics are usually made of Meta-aramid, Para-aramid, Polybenzimidazole, and fire retardant cotton. Changes in the tensile strength of the high-performance fabrics used in bunker gear upon being exposed to radiant heat have been assessed. Different amounts of moisture were added into the fabric system being exposed to heat. The combination of which simulates the wearers’ sweating.

### 2.1. Materials

**Figure 1:**
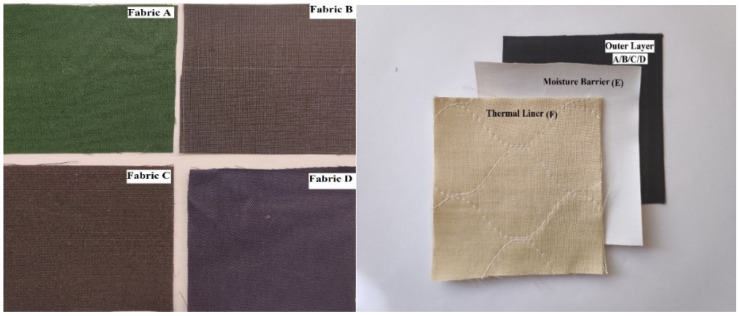
Fabric A, B, C, D, and an arrangement of a three-layered fabric system.

Four different types of high-performance fabrics A, B, C, and D were selected which are typically used in firefighters’ bunker gear. For the single-layer fabric system these four different high-performance fabrics were used. To compose the three-layered fabric system, a moisture barrier (fabric E) and thermal liner (fabric F) were used together with these four high-performance fabrics. Details of fabrics A, B, C, D, E, F are given in Table 1. Four different multilayered assemblies were tested: AEF, BEF, CEF, and DEF. The four different high-performance fabrics and three-layered fabric systems are shown in Figure 1. The tensile strength of the selected fabrics in dry and wet conditions was measured by using a tensile strength tester (SDL Atlas, Rock Hill, SC, USA) before and after exposure to radiant heat. The process of moisture application, heat exposure and tensile strength measurement are discussed below.

The properties of all four high-performance fabrics are listed in Table 1.

### 2.2. Moisture Application

Two different scenarios for moisture application were followed. Moisture was applied to the fabrics (A, B, C, and D) to simulate the wearers’ sweating during the heat exposure in the single-layer fabric system. However, moisture was applied on the thermal liner to simulate the sweat of the wearers in three-layered fabric systems. For the single layer, the weight of the dry fabric samples was measured first, then the necessary amount of distilled water was sprayed on the fabric surface until the added moisture content reached 20%, 50%, and 100%, respectively. If the fabric weight was X gram 20%, 50%, and 100% moisture indicated that the final weight of the fabric after moisture addition is 1.2×, 1.5×, and 2×. For the three-layered fabric system, the weight of the dry sample (three layers together) was measured first, then with the help of a dropper, the required amount of distilled water was added to the thermal liner. Water was added by spray or dropper until the weight balance showed the required weight. By using the dropper, it was possible to apply the water all over the thermal liner uniformly. The amount of water needed to be added in the single-layer fabric system was comparatively lower than the water added in the three-layered fabric system. A spray was used to ensure the even distribution of the small amount of water on the single-layer system fabric and a dropper was used to apply water in the three-layered fabric system.

**Figure 2:**
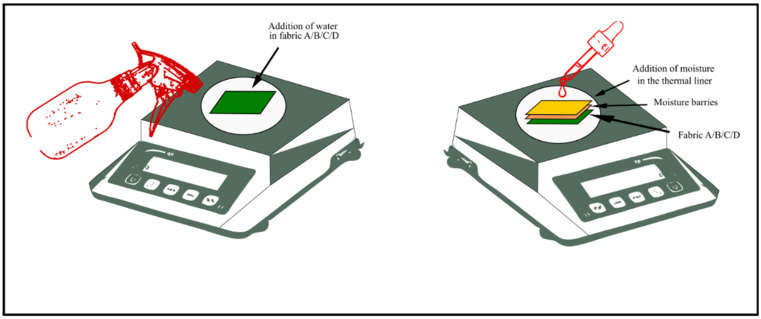
Schematic diagram of addition of water in the fabrics by spray and dropper.

Distance between the fabric and the spray nozzle was approximately 6 inches to ensure that water droplets fell over the whole fabric evenly. Water was added to the thermal liner (three-layered fabric system) by using a dropper. Drops of water were added in different sections (four corners, center, middle of the four sides) of the fabric to ensure even distribution of the water in the fabric. Figure 2 shows the application of water in fabrics A, B, C, and D in single layer system and also in the thermal liner of the three-layered fabric system to simulate the firefighter’s sweating. Fabrics were treated in three different moisture content levels: 20%, 50%, and 100%. Samples were left on the weight balance for five minutes before being exposed to heat to ensure that the required amount of moisture has distributed throughout the fabric. In this period, the sample remained on the weight balance and the final weight was checked before the exposure to ensure the required amount of water was absorbed in the fabrics.

### 2.3. Cone Calorimeter

**Figure 3:**
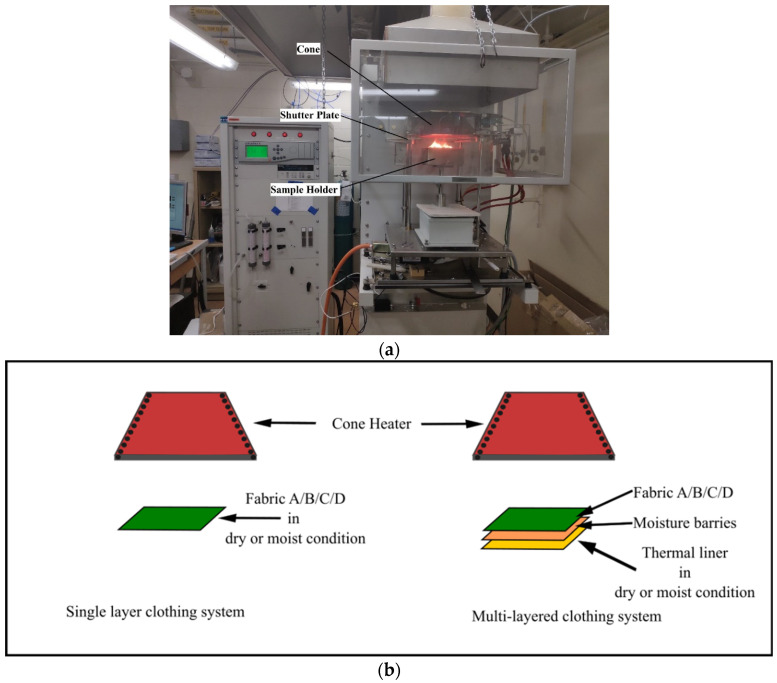
(**a**) Cone calorimeter. (**b**) Schematic diagram of heat exposure of single and multi-layered fabric system.

To simulate the radiant heat exposure that the firefighters face during fire activities, a cone calorimeter (Fire Testing Technology Ltd., East Grinstead, United Kingdom) (Figure 3a) was used. The cone calorimeter can provide constant amount of heat on a relatively small size sample (typically 10 cm by 10 cm) [36]. Therefore, the cone calorimeter is the most widely used instrument to study fire-related behavior of materials [37]. The cone calorimeter is named after the conical shape of the radiant heater; with a 160 mm diameter at the bottom and 80 mm on top, the cone produces nearly uniform heat flux on the sample under study [38]. The radiant heat flux can be controlled by the cone-shaped heating element, which is made out of Inconel alloys [39]. These alloys are suitable for extreme heat environments due to their oxidation-corrosion resistance properties [40]. The specimen holder and the heater are placed horizontally, and there is a shutter plate in between separating the fabric from the heat. The test begins when the plate is removed letting the radiant heat reach the surface of the surface.

Fabric samples of (10 cm by 10 cm) were prepared according to the standard (ASTM E1354) and placed 25 mm below the cone heater (Figure 3b) [38]. The samples were exposed to three different heat flux levels: 10 kW/m^2^, 15 kW/m^2^, and 20 kW/m^2^ for five minutes. To investigate the effect of different heat flux on the mechanical properties, all the fabrics were exposed to the heat for the same amount of time. To keep it constant, all four fabrics were exposed to different level of heat flux for five minutes. At 20 kW/m^2^ more than five minutes of exposure caused complete burn out of fabric D. Five minutes exposure was the highest exposure time without complete degradation of the fabrics; therefore, measuring the strength of the fabrics was possible. Standard test method ASTM E1354 was designed to evaluate fire-retardant materials. Following this test method, samples were conditioned in the textile lab for 24 hours before the heat exposure. After conditioning, samples were transferred to zip-top bags and exposed to radiant heat within three hours. The moisture was applied to the fabrics just before the heat exposure. The details of moisture application are explained in Section 2.2. Once the exposure was completed, samples were immediately transferred to the zip-top bags and again conditioned for 24 h before measuring the strength. The heat exposure time was five minutes for all the samples.

### 2.4. Tensile Strength Tester

**Figure 4:**
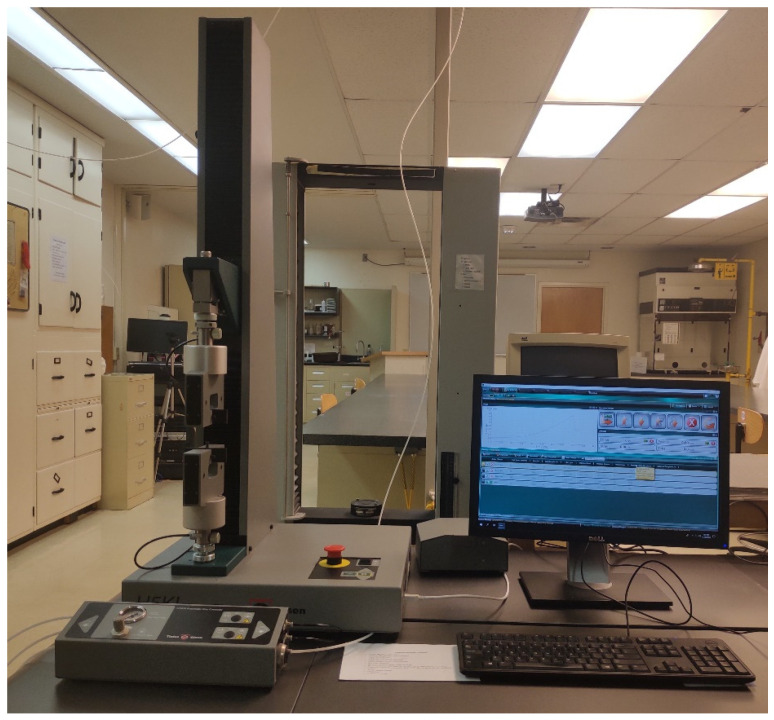
Tensile strength tester.

Tinius Olsen H5K tensile strength tester (Figure 4) (SDL Atlas, Rock Hill, SC, USA) was used to measure the tensile strength (in Newton N) of the fabric before and after radiant heat exposure. ASTM D5034 Grab Test method was used to measure the tensile strength of the fabric [35]. The Grab test method was chosen over the Strip test method [41] since it is mentioned that strip testing sometimes may lead to clamp fractures. The same research also concluded that for testing textile fabric, the grab test is sufficient [42]. For the tensile strength test, the distance between the jaws was 40 mm, and tensile speed was 50 mm/min. The tensile strength of the fabrics was measured in the warp direction only. The required tensile strength of warp yarn is usually higher than the weft yarn due to the weaving mechanism. Therefore, the strength of woven fabric in the warp direction is usually higher than in the weft direction. If the warp yarn does not satisfy the minimum requirement after the heat exposure, then it can be said that weft will not satisfy either, since the tensile strength of weft is lower than the warp. Therefore, the tensile strength was measured only in the warp direction in this study.

Tensile strength of fabrics A, B, C, and D was measured after each fabric was exposed to moisture and heat as part of a single-layered system (the fabric itself) and as part of multi-layered systems AEF, BEF, CEF, and DEF, respectively. In single-layer fabric system fabrics, A, B, C, and D were exposed to different radiant heat levels in dry and moist conditions. After the five minutes of heat exposure, samples were immediately placed in zip-top bags. Before measuring the tensile strength, all samples were conditioned in the textile lab for 24 hours. Similarly, in the three-layered fabric system, AEF, BEF, CEF, and DEF fabric samples were exposed to radiant heat. Fabric combinations were exposed either in dry condition or with the presence of moisture in the thermal liner. Similarly, after the five minutes of heat exposure, samples were secured in zip-top bags. The outer layer was then removed from the three-layered system and conditioned for 24 hours measuring the tensile strength.

### 2.5. Test Protocol

In total, 16 different testing scenarios were created for each of the single and three-layered combinations in each heat flux level. Each testing was repeated three times, therefore there were forty-eight samples in each fabric combination. Each fabric combination was exposed to three different heat exposure levels 10 kW/m^2^, 15 kW/m^2^, and 20 kW/m^2^. For each exposure level, there were four different moisture levels. Details of test scenarios are given in Table 2. Three repetitions of the test were conducted in each scenario.

### 2.6. Analyzing the Experimental Data

The data were analyzed using the SPSS Statistics Analysis tool and were categorized into three groups. In the first group, the tensile strength data of all four different performance fabrics A, B, C, and D in dry conditions were analyzed. The tensile strength of all four fabrics exposed at three different heat flux falls under this group. The properties of the fabrics (i.e., weight/unit length, thickness, fabric count) and the heat flux intensities were the independent variables here the tensile strength was the dependent variable. In the second group, a single-layer fabric system with the presence of moisture was analyzed. Under this category, the amount of added moisture and the heat flux intensities were the independent variables along with the above-mentioned fabric properties (i.e., weight/unit length, thickness, fabric count) the tensile strength was the dependent variable. The three-layered fabric systems where the moisture was added in the thermal liner falls under the third category. Variables in the third group are the same as the second group, where the only difference is that the multi-layer fabric system was analyzed within this group instead of the single-layer fabric system, where moisture was applied in the thermal liner fabrics. Properties (i.e., weight/unit length, thickness, fabric count) of the three-layered fabric systems and tensile strength of the outer layer have been normalized between -1 and +1, while the average value is set to zero. The normalized variable *X _i,norm_* is expressed in the below equation. The normalization process reduces the redundancy rates in the data by pulling out the abnormal factors.

(1) $$X_{i,norm}=\frac{X_{i}-X_{i,avg}}{R_{i,max}}$$

where, *R_i,max_* = Maximum [(*X_i,max_* − *X_i,avg_*),(*X_i,avg_* − *X_i,min_*)]. In the above equation the *X_i_* is the value of selected variable (thickness, air permeability, thermal and evaporative resistance, and tensile strength), *X_i,avg_* is the average value of that particular variable, *X_i,min_* is the minimum value of that variable, *X_i,max_* is the maximum value of that variable, and *R_i,max_* is the maximum range between the average value and either the minimum or the maximum of that variable. A multi-linear regression analysis of the normalized dataset of the fabric properties and the tensile strength has been conducted by using the SPSS Statistics Analysis tool to understand the relation between the fabric properties and the change in tensile strength. It has been hypothesized that these fabric properties can represent linear regression with tensile strength. Different studies were found where linear regression analysis was used to model the relation between fabric properties and performance [43,44]. The amount of moisture added, and the heat intensity levels were considered as the ordinal independent variables for the regression analysis. Among the three independent variables (i.e., weight, thickness, and fabric count), the properties that showed the highest absolute regression coefficient was considered the key property affecting the tensile strength. This analysis was carried out at 95% Confidence Interval. *p*-value obtained from regression analysis was analyzed to identify the fabric properties that have a significant effect on the tensile strength loss. The significance test was carried out at 0.05 significance level. Thus, if the obtained value was less than 0.05 then the properties were significant.

## 3. Results and Discussion

Section 3 are divided into three sub-sections. In Section 3.1, change of tensile strength of the fabrics A, B, C, and D in the dry condition is discussed. In Section 3.2, the tensile strength change of the fabrics A, B, C, and D is discussed while only single layer fabrics were exposed with moisture. In Section 3.3, changes of tensile strength of the outer layers in three-layered fabric systems are discussed while the fabric system was exposed with moisture in the thermal liner. The radiant heat-treated fabrics were conditioned for 24 h before measuring the tensile strength. The tensile strength (warp direction) of the fabrics A, B, C, and D were measured by the tensile strength tester using the standard method ASTM D5034.

### 3.1. Effect of Radiant Heat on Tensile Strength of the Fabrics in Dry Condition

The initial strength of the fabric usually depends on the fabric and yarn properties, such as count, twist, ends and picks per inch, cover factor, weave structure, etc., and the type of fiber present in the fabric [45,46,47,48,49]. The summary of the radiant heat on tensile strength summarizes in Table 3. Since three of the experimented fabrics (A, B, and C) were made from synthetic fiber and fabric D was made from a natural fiber, the initial tensile strength of these two categories of fabrics was significantly different. In addition, the changing behavior of tensile strength after the radiant heat exposure also can be categorized into two groups. fabrics A, B, C behave similarly compared to fabric D, which behaved completely differently. In dry conditions, the level of radiant heat flux for an exposure time of five minutes has a significant (*p* < 0.05) effect on the tensile strengths of the fabric used (Table 3). In these five minutes of exposure, with the increase in heat flux intensity, the strength of the fabrics decreased. The loss of strength was higher at 15 and 20 kW/m^2^ heat flux compared to the 10 kW/m^2^ heat flux. Minimum loss of strength was around 50% and 75% at 15 kW/m^2^ and 20 kW/m^2^ heat flux, respectively, which was only around 4% at 10 kW/m^2^. All four fabrics showed a similar trend of strength loss, only strength loss of fabric D was severe compared to the others. The difference between fabric D the fabrics A, B, and C is due to the type of fiber present in the fabrics. In general, natural fibers have lower strength compared to synthetic fibers [50]. The tensile strength mostly depends on the crystallinity and spiral angle of the polymers. Higher crystallinity and lower spiral angle in general give higher strength [51]. Usually, cotton has a lower crystallinity than synthetic fibers, moreover, the spiral angle of the polymers in cotton fiber is around or more than 20 degrees [51,52].

**Figure 5:**
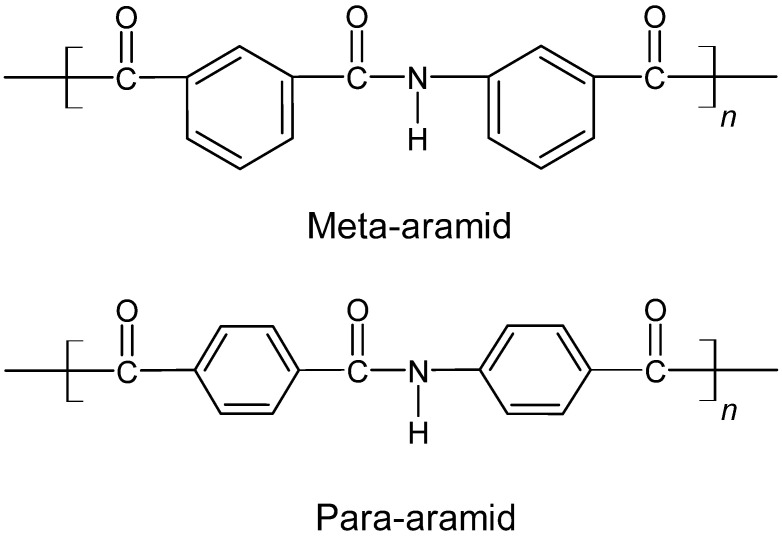
Structures of Meta-Aramid and Para-Aramid fibers.

The tensile strength loss of fabric A was much higher at 20 kW/m^2^ compared to fabrics B and C. The reason that Fabric B and C showed higher resistance in tensile strength loss compared to fabric A lies in their polymer structure. The fabric B and C which is made from para-aramid blend fiber connects at the para-position of the phenyl link, whereas the fabric A meta-aramid fibers connect at the meta-position. Therefore, polymers in para-aramid fiber are highly compact compared to the meta-aramid fibers [53,54]. Due to the lower compactness of the meta-aramid fiber compared to the para-aramid fiber, the meta-aramid fiber is not as strong as para-aramid fiber and is also more flexible than the para-aramid fiber [53,54]. Figure 5 shows the polymer structure of both meta-aramid and para-aramid fibers.

**Figure 6:**
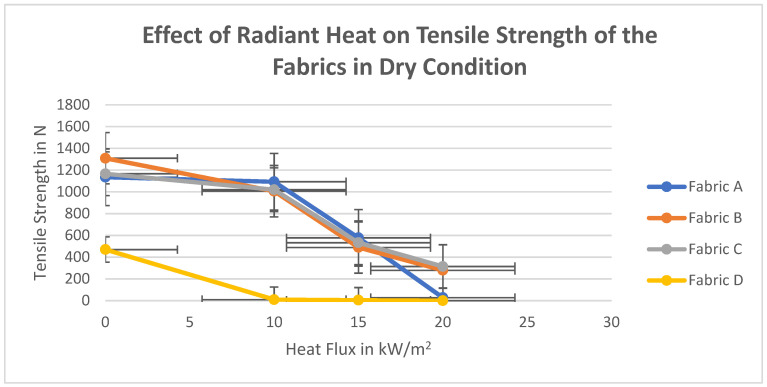
Effect of radiant heat on tensile strength of the fabrics in dry condition.

Effect of radiant heat exposure in dry condition on tensile strength of all four outer layers has been illustrated in Figure 6.

The tensile strength of the fabric is mostly dependent on the organization of the polymer chains and the macrostructure [55,56,57]. A similar pattern of loss of tensile strength with increased temperature is observed from Figure 6. The loss of tensile strength can be explained due to the fibrillar to the lamellar transformations within the fibers which cause an increase in crystallinity with lamellar spacing [58]. A linear regression analysis tool has been used to find out the R square and *t*-test (*t* and *p*) values. As mentioned earlier, data have been grouped into three categories. In the first category, the tensile strength of four high-performance fabrics exposed in three different heat flux in dry conditions have been analyzed. Independent variables: (i) fabric properties (Weight/unit length, thickness, fabric count); (ii) heat flux intensities (10, 15, and 20 kW/m^2^) are the ordinal variables. Dependent variable: tensile strength of the fabrics A, B, C, and D. The results are as shown in Table 4.

The *t*-test value matches the earlier discussion. The negative *t* value of the heat intensity levels indicated that an increase in heat flux reduces the tensile strength of the fabric. The *p*-value suggests the significance of the effect on tensile strength. From the R square values, it can be seen that thickness has the highest value compared to linear density and fabric count. Therefore, it can be said that thickness is the most important property while considering the tensile strength of the fabric [59]. Fabric count seems the second most important property. Nevertheless, both thickness and fabric count moderately affect the tensile strength as their R square values are fairly high [58,60].

### 3.2. Effect of Moisture and Radiant Heat on Tensile Strength of Fabrics in Single Layer Fabric System

**Figure 7:**
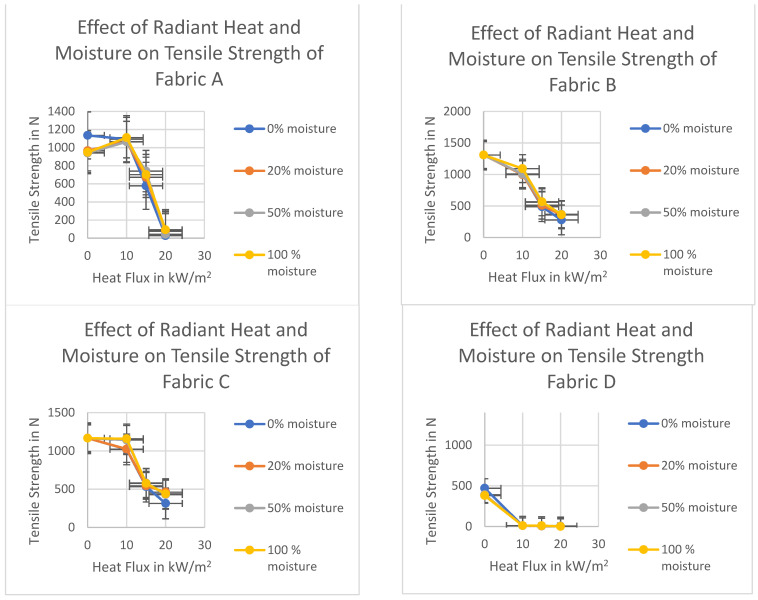
Effect of moisture and radiant heat on tensile strength of fabrics in single layer fabric system.

In the single-layer fabric system, moisture did not have much effect on the tensile strength of the fabrics (Figure 7). The tensile strength of fabrics in the single-layer fabric system at both dry and moist conditions was almost similar. The addition of moisture in the single-layer fabric system slightly affected the tensile strength loss. This is because of the ease of evaporation of the water from the single-layer fabric. The moisture of the outer layer evaporated very quickly, which resulted in increased temperature in the fabric system, leading to the fabric to behave similarly to the dry fabric [61]. The quick evaporation of the moisture results in the thermal degradation of the polymer chain. Since the moisture evaporated very quickly, the tensile strength of the moist fabric was almost like the dry fabric. A slightly improved tensile strength was shown at 10 kW/m^2^ when the moisture percentage was 100%. The tensile strength of fabric A increased initially with respect to moisture during the heat exposure at 10 kW/m^2^. This increasing tensile strength phenomenon could be explained based on the initial strength of fabric A in moist conditions. The initial tensile strength of fabric A in moist condition was lower than the dry condition. This is likely due to moisture reducing the friction between the fibers which resulted in lower tensile strength of the fabric in moist conditions before the heat exposure.

Multiple linear regression analysis tool has been used to determine the R square and *t*-test value (*t* and *p* values). The independent and dependent variables are as follows: Independent variables: (i) Fabric Properties (Weight/unit length, thickness, fabric count); (ii) Heat flux intensities (0, 10, 15, and 20 kW/m^2^), and moisture addition amount (0, 20, 50, and 100%) are the ordinal independent variables. Dependent variable: Tensile strength of the outer layer fabric. The results are shown in Table 5. All four fabrics A, B, C, and D behaved almost similarly in both dry and moist conditions in single-layer fabric system. Only a very minor difference was seen when moisture addition was 100%.

In the single-layered fabric system, the moisture had no or minimal effect on the tensile strength. The statistical values also suggest a similar result. The *t*-values for the moisture are positive and almost near zero. This suggests that moisture in the single-layer has a minimum effect on the tensile strength of the outer layer fabric. On the other hand, heat flux intensity has a similar negative effect on the *t*-value. From the R square values, it can be seen that thickness has the highest value compared to linear density and fabric count. Therefore, it can be said that thickness is the most important property while considering the tensile strength of the fabric. Fabric count seems the second most important property. Nevertheless, both thickness and fabric count moderately affect the tensile strength as their R square values are fairly high. The summary of the effects of moisture and radiant heat in single-layer fabric system is shown in the table below (Table 6).

### 3.3. Effect of Moisture and Radiant Heat on Tensile Strength of Outer Layer Fabrics in Three-Layered Fabric System

**Figure 8:**
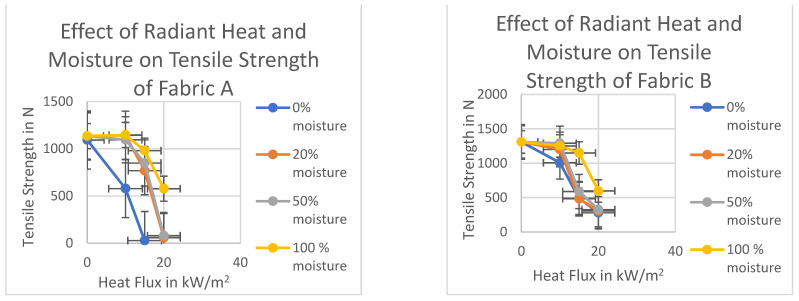

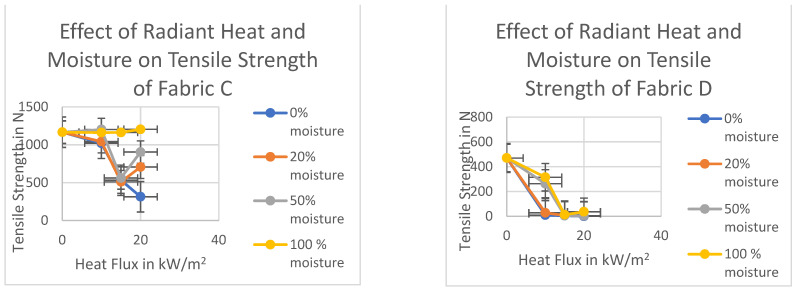
Effect of radiant heat and moisture on tensile strength of the outer layer fabrics in multi-layered fabric system.

Added moisture had a significant (*p* < 0.05) positive effect on the strength loss of the fabrics (Figure 8). At a lower heat intensity level of 10 kW/m^2^ and five minutes of exposure time, the tensile strength loss percentage was very low compared to the dry and single layer moist fabrics. No or minimum strength loss was seen for most of the fabrics at lower heat flux with the presence of moisture in the thermal liner. With the increase in heat flux, the effect of moisture decreased eventually. At 15 kW/m^2^ heat flux 100% moisture showed the highest effect on the tensile strength loss. Only without the fabric D all other fabrics were able to retain most of their strength when exposed to 15 kW/m^2^ heat flux for five minutes. The highest amount of strength loss was only 15%, which was around 60% at 15 kW/m^2^ without moisture or the presence of moisture in the single layer.

At lower temperatures in the presence of moisture, there might be orientation changes of polymer chain occurred in some fabrics, which led to an increase in the crystalline region and increased the strength of the fiber. The strength loss was also lower at 15 kW/m^2^ and 20 kW/m^2^ in the presence of moisture compared to the dry fabrics for most of the fabrics. With moisture increasing, the strength loss percentage decreased. At higher heat flux, lower moisture content (20% and 50%) did not affect the tensile strength significantly. The moisture helped significantly to retain the tensile strength, especially at lower temperatures. The heat is absorbed in the process of transforming moisture into vapor. Since most of the heat energy has been used to evaporate the moisture the temperature inside of the exposed samples did not increase much [62]. Therefore, the loss of tensile strength was considerably lower than dry and single layer moist conditions. The presence of moisture in the thermal liner could increase the heat capacity of the fabrics, which resulted in a significant amount of thermal energy storage within the fabric system [6,59,63,64].

Addition of moisture in the thermal liner had a significant positive effect on the tensile strength of fabrics A, B, and C, with only fabric D being the exception. This phenomenon is due to the type of fiber (i.e., natural or synthetic) present in the fabric, which has been discussed earlier. Added moisture had a significant effect on the strength loss of Fabric A. In wet condition, there is negligible amount of strength loss or slight gain in strength shown by this fiber. Tensile strength data show that the strength loss percentage was negative for 20% and 100% moisture addition. The strength loss was also significantly lower at 15 kW/m^2^ and 20 kW/m^2^ with the presence of moisture compared to the dry fabrics. With the increased moisture, the strength loss percentage decreased. The strength losses were 32%, 25%, and 14%, respectively, for 20%, 50%, and 100% moisture addition at 15 kW/m^2^. However, at the highest radiant flux at 20 kW/m^2^ the strength loss percentages were 95% and 93% for 20% and 50% moisture addition. At higher heat flux, lower moisture content (20% and 50%) did not affect the tensile strength significantly. However, at 100% moisture addition the heat loss percentage was only 49% which is half compared to 98% at the dry condition at 20 kW/m^2^. The 20% and 50% moisture addition had a very minor effect on the tensile strength at 15 and 20 kW/m^2^ in fabric B. The difference was below 10% at this moisture addition compared to the dry condition. However, the 100% moisture addition showed a significant effect even at higher heat flux. The tensile strength loss was 12% and 54% at 15 and 20 kW/m^2^, respectively, which were 63% and 79% for the same fabric in dry condition.

During the five minutes of exposure after the evaporation of the 20% and 50% added moisture maybe there was sufficient time to degrade the outer layer fabric. Therefore, this amount of moisture did not help the fabric retain its tensile strength by increasing the heat capacity of the fabric. However, the 100% moisture addition increased the heat capacity of the fabric to a certain level that the fabric to retain its strength, and therefore the tensile strength loss was lower at this moisture content. At lower radiant heat 10 kW/m^2^ and with 20% moisture addition, the strength loss was 11% which was 13% at dry condition for the fabric C. Therefore, 20% moisture did not help significantly at lower heat flux. The increased heat capacity of the fabric for 20% moisture addition was not sufficient enough to retain the tensile strength during five minutes of exposure. However, for the 50% and 100% moisture addition, there was no loss or increase in the tensile strength (Figure 8). Therefore, the amount of moisture can play a significant effect on the tensile strength at low radiant heat. At 20% and 50% moisture, there was no effect at 20 kW/m^2^ heat exposure. The heat loss percentage was same for the 20% and 50% moisture while compared to the dry fabric. However, 100% moisture played a significant role at 20 kW/m^2^. At a higher heat flux of 20 kW/m^2^ this fabric behaved very differently compared to the other fabrics. The strength loss was lower at 20 kW/m^2^ compared to the 15 kW/m^2^. At 100% moisture content and 20 kW/m^2^ radiant heat this fabric behaved similarly to the 50% moisture content and 10 kW/m^2^ radiant heat exposure, which is a slight increase in tensile strength. Moisture played a significant role at higher heat flux 20 kW/m^2^. The change of orientation of the polymer chains in presence of moisture could be the reason for this increase in strength. The strength loss decreased to 39% with 20% moisture addition which was 73% at dry conditions. This then comes down to 22% loss at 50% moisture, and then 3% increase at 100% moisture addition.

As discussed above, the least resistance to radiant heat exposure is shown by fabric D (Figure 8). In dry condition, this fabric lost almost 100% of its strength even at 10 kW/m^2^. The moisture had a positive effect only at the lower radiant heat exposure 10 kW/m^2^. The tensile strength loss was 44% and 33%, respectively for 50% and 100% moisture content compared to the 98% at dry conditions. However, at higher heat flux 15 and 20 kW/m^2^ moisture did not play any significant role. The strength loss of the moist fabrics at all percentages was similar to the dry fabrics. Moisture did not help much at higher temperatures because once the moisture evaporated the temperature inside the sample raised during five minutes of exposure [65]. Fabric D loses its strength even at a lower heat flux of 10 kW/m^2^. Therefore, at 15 and 20 kW/m^2^ moisture could not help much in retaining the tensile strength. Once the moisture evaporated, the temperature increased, and fabric lost its tensile strength immediately. Multi-layer fabric system with the presence of moisture in the thermal liner during the exposure has been analyzed. Same linear regression analysis tool has been used to determine the R square and *t*-test value (*t* and *p* values). The independent and dependent variables are as follows: Independent variables: (i) Fabric properties (Weight/unit length, thickness, fabric count); (ii) Heat flux intensities (0, 10, 15, and 20 kW/m^2^) and moisture addition amount (0, 20, 50, and 100%) are the ordinal independent variables. Dependent variable: Tensile strength of the outer layer fabric. The results are shown in Table 7.

From the earlier discussion, we have seen that in the three-layered fabric system with the presence of moisture in the thermal liner, moisture has positive effect on the tensile strength. On the other hand, the level of heat intensity has a negative effect on the tensile strength. The *t*-test value in the above table shows the same result. For all four outer layers, *t*-values for moisture have a positive value, and heat intensity values have a negative value. All the *t*-test values except fabric count are statistically significant when the alpha value is 0.1 or lower.

Additionally, the R^2^ values of the multi-layer fabric system are lower compared to the single-layer fabric system. This suggests the moisture in the thermal liner plays crucial part in determining the effect on tensile strength of the fabrics compared to the presence of moisture in the single layer. Since moisture in the outer layer did not affect the tensile strength, the strength almost depended on the heat flux intensities solely. Therefore, the R square values are greater in the single-layered fabric system compared to the three-layered fabric system. Similar to the previous discussion, thickness is the most important property when we consider the fabric tensile strength. Similarly, fabric count is the second most important property. Nevertheless, both thickness and fabric count moderately affect the tensile strength as their R square values are fairly high. Table 8 summarizes the combined effect of radiant heat and moisture on the tensile strength of all four outer layer fabrics.

## 4. Conclusions

This study investigated the change of tensile strength of the fabrics in presence of moisture, used in firefighters’ bunker gear under radiant heat exposure. The tensile strength of the fabrics has been characterized under different heat flux in dry and moist conditions. Thickness and the fabric count have been found as the key fabric properties affecting the tensile strength. The presence of moisture also plays a complicated role in determining the change of tensile strength depending on the amount and location of the moisture. Moisture had a significant positive effect on the tensile strength when the moisture was in the thermal liner and the amount of moisture was higher. This research will help to understand the overall integrity of the firefighters’ bunker gear after being worn at fire sites. However, the change of tensile strength may not be directly related to the protective performance. Therefore, further research may be completed to develop the relationship between the change of tensile strength and the protective performance of thermal protective clothing. The changes of tensile strength also could not be quantified in terms of orientational changes of polymer chains, which could be another interesting study for further development of this research. It is expected that this research will help to understand the change of tensile strength of the fabric used in thermal protective clothing after being exposed to radiant heat, which will eventually help to identify the overall integrity of the firefighters’ thermal protective clothing. Our research will help to develop improved fabrics by maintaining better integrity of the firefighters’ clothing while on duty. This effort could help to improve occupational health and safety for firefighters

## Figures and Tables

**Table 1:** Properties of the high-performance fabrics experimented.

Sample	Fiber Content	Weave Structure	Weight ^a^ (g/m^2^)	Thickness ^b^ (mm)	EPI/PPI, Fabric Count ^c^ (EPI + PPI)	Absorbency ^d^	Tensile Strength ^e^ (Warp Direction)
A	Meta-aramid	Twill	261	0.62	75/45 (120)	0	1135 N
B	Para-aramid/Meta-aramid	Ripstop	204	0.53	60/45 (105)	100	1309 N
C	Polybenzimidazole/Para-aramid	Twill	196	0.51	45/45 (90)	100	1166 N
D	FR Cotton	Twill	269	0.71	90/50 (140)	0	470 N
E	Polybenzimidazole/Para-aramid/Meta-aramid	Non-woven	202	0.36	NA	100	NA
F	Aramid/FR Cotton/Polyamide	Woven facecloth/Non-woven batting	225	1.44	NA	0	NA

^a^ Measured according to ASTM D3776 [31]; ^b^ Measured according to ASTM D1777 [32]; ^c^ Measured according to ASTM D3775 [33]; ^d^ Measured according to AATCC 22 [34]; ^e^ Measured according to ASTM D5034 [35].

**Table 2:** Details of test scenarios.

Fabric System	Heat Flux	Fabric Combination	Moisture Addition	Time of Heat Exposure	Test Scenarios
Single Layer	0 kW/m^2^	A	0/20/50/100%	5 min	4
		B			4
		C			4
		D			4
	10 kW/m^2^	A	0/20/50/100%	5 min	4
		B			4
		C			4
		D			4
	15 kW/m^2^	A	0/20/50/100%	5 min	4
		B			4
		C			4
		D			4
	20 kW/m^2^	A	0/20/50/100%	5 min	4
		B			4
		C			4
		D			4
Multi-layers	0 kW/m^2^	AEF	0/20/50/100%	5 min	4
		BEF			4
		CEF			4
		DEF			4
	10 kW/m^2^	AEF	0/20/50/100%	5 min	4
		BEF			4
		CEF			4
		DEF			4
	15 kW/m^2^	AEF	0/20/50/100%	5 min	4
		BEF			4
		CEF			4
		DEF			4
	20 kW/m^2^	AEF	0/20/50/100%	5 min	4
		BEF			4
		CEF			4
		DEF			4

**Table 3:** Effect of radiant heat on tensile strength (Dry condition).

Samples	Initial Strength	Heat Flux					
		10 kW/m^2^	Tensile Strength Loss %	15 kW/m^2^	Tensile Strength Loss %	20 kW/m^2^	Tensile Strength Loss %
		0% Moisture		0% Moisture		0% Moisture	
Fabric A	1135 N	1093 N	3.7%	578 N	49%	28 N	98%
Fabric B	1309 N	1006 N	23%	489 N	63%	279 N	79%
Fabric C	1166 N	1020 N	13%	533 N	54%	314 N	73%
Fabric D	470 N	9 N	98%	4.4 N	99%	0 N	100%

**Table 4:** Statistical analysis of the tensile strength of the outer layer fabrics exposed in dry conditions.

Model Summary		
R^2^ Value	*F*	*p*
0.83	8.82	0.007
Coefficients	*t*	*p*
Weight/unit Length	2.21	0.063
Thickness	−1.92	0.096
Fabric Count	0.102	0.921
Heat Intensity Level	−3.790	0.007
Individual R Square Values between the Fabric Properties and Tensile Strength		
Fabric Properties	R^2^ Value	
Weight/unit length	0.225	
Thickness	0.379	
Fabric Count	0.358	

**Table 5:** Statistical analysis of single layer fabric system in presence of moisture.

Model Summary		
R Square	*F*	*p*
0.84	30	0.001
Coefficients	*t*	*p*
Weight/unit length	4.415	0.000
Thickness	−3.642	0.001
Fabric Count	0.037	0.970
Heat Intensity Level	−7.97	0.0001
Moisture Level	0.431	0.670
Individual R Square Values between the Fabric Properties and Tensile Strength		
Fabric Properties	R Square Value	
Weight/unit length	0.245	
Thickness	0.392	
Fabric Count	0.374	

**Table 6:** Effect of moisture and radiant heat on tensile strength of outer layer fabrics (Single layer).

Samples	Heat Flux																							
	10 kW/m^2^								15 kW/m^2^								20 kW/m^2^							
									Moisture Addition and Tensile Strength Loss %															
	0%	Δ%	20%	Δ%	50%	Δ%	100%	Δ%	0%	Δ%	20%	Δ%	50%	Δ%	100%	Δ%	0%	Δ%	20%	Δ%	50%	Δ%	100%	Δ%
	Strength (N)		Strength (N)		Strength (N)		Strength (N)		Strength (N)		Strength (N)		Strength (N)		Strength (N)		Strength (N)		Strength (N)		Strength (N)		Strength (N)	
Fabric A	1093	3.7	1067	−10	1064	−13	1110	−17	578	49	672	30	741	21	702	26	28	98	77	92	42	96	93	90
Fabric B	1006	23	997	23.80	1007	23	7093	16.5	489	63	512	61	559	57	568	57	279	79	359	73	369	72	363	72
Fabric C	1020	13	1024	12	1142	3	1159	0.60	533	54	544	53	581	50	575	51	314	73	461	60	429	63	439	62
Fabric D	9	98	9.6	98	8.4	97.8	10.6	97.1	4.4	99	5	98.7	6.8	98.3	9.2	97.6	0	100	4.5	99	4.7	99	0	100

**Table 7:** Statistical analysis of multi-layered fabric system in presence of moisture in the thermal liner.

Model Summary		
R Square	*F*	*p*
0.81	26.02	0.001
Coefficients	*t*	*p*
Weight/unit length	3.434	0.002
Thickness	−2.703	0.011
Fabric Count	−0.642	0.526
Heat Intensity Level	−5.941	0.0001
Moisture Level	3.214	0.003
Individual R Square Values between the Fabric Properties and Tensile Strength		
Fabric Properties	R Square Value	
Weight/unit length	0.303	
Thickness	0.452	
Fabric Count	0.446	

**Table 8:** Effect of radiant heat on tensile strength (Wet condition).

Samples	Heat Flux																							
	10 kW/m^2^								15 kW/m^2^								20 kW/m^2^							
	Moisture Addition and Tensile Strength Loss %																							
	0%	Δ%	20%	Δ%	50%	Δ%	100%	Δ%	0%	Δ%	20%	Δ%	50%	Δ%	100%	Δ%	0%	Δ%	20%	Δ%	50%	Δ%	100%	Δ%
	Strength (N)		Strength (N)		Strength (N)		Strength (N)		Strength (N)		Strength (N)		Strength (N)		Strength (N)		Strength (N)		Strength (N)		Strength (N)		Strength (N)	
Fabric A	1093	3.7	1143	−0.7	1095	3.5	1146	−0.97	578	49	768	32	849	25	980	14	28	98	58	95	80	93	577	49
Fabric B	1006	23	1200	8.3	1291	1.4	1251	4.4	489	63	483	63	588	55	1149	12	279	79	304	77	325	75	596	54
Fabric C	1020	13	1042	11	1202	−3	1161	0.4	533	54	511	56	562	52	1163	0.3	314	73	707	39	904	22	1204	−3.3
Fabric D	9	98	28	94	263	44	315	33	4.4	99	5.3	98.9	5.1	98.9	12.8	97	0	100	4.2	99	3.3	99	36.5	92

Δ: Change of tensile strength (compared to the initial strength before the heat exposure and dry condition).

## Data Availability

The data presented in this study are available on request from the corresponding author.
